# Compound heterozygous variants within two conserved sialyltransferase motifs of *ST3GAL5* cause GM3 synthase deficiency

**DOI:** 10.1002/jmd2.12353

**Published:** 2022-11-29

**Authors:** Natasha Rudy, Kazuhiro Aoki, Amitha Ananth, Lynda Holloway, Cindy Skinner, Anna Hurst, Michael Tiemeyer, Richard Steet

**Affiliations:** ^1^ Department of Genetics The University of Alabama at Birmingham Birmingham Alabama USA; ^2^ Complex Carbohydrate Research Center University of Georgia Athens Georgia USA; ^3^ Greenwood Genetic Center Greenwood South Carolina USA

**Keywords:** GM3, GM3 synthase deficiency, ST3GAL5, sialyltransferase

## Abstract

GM3 synthase deficiency (GM3SD) is caused by biallelic variants in *ST3GAL5*. The ganglioside GM3, enriched in neuronal tissues, is a component of lipid rafts and regulates numerous signaling pathways. Affected individuals with GM3SD exhibit global developmental delay, progressive microcephaly, and dyskinetic movements. Hearing loss and altered skin pigmentation are also common. Most of the reported variants in *ST3GAL5* are found in motifs conserved across all sialyltransferases within the GT29 family of enzymes. These motifs include motif L and motif S which contain amino acids responsible for substrate binding. These loss‐of‐function variants cause greatly reduced biosynthesis of GM3 and gangliosides derived from GM3. Here we describe an affected female with typical GM3SD features bearing two novel variants that reside in the other two conserved sialyltransferase motifs (motif 3 and motif VS). These missense alterations occur in amino acid residues that are strictly invariant across the entire GT29 family of sialyltransferases. The functional significance of these variants was confirmed by mass spectrometric analysis of plasma glycolipids, demonstrating a striking loss of GM3 and accumulation of lactosylceramide and Gb3 in the patient. The glycolipid profile changes were accompanied by an increase in ceramide chain length on LacCer. No changes in receptor tyrosine phosphorylation were observed in patient‐derived lymphoblasts, indicating that GM3 synthase loss‐of‐function in this cell type does not impact receptor tyrosine kinase activity. These findings demonstrate the high prevalence of loss‐of‐function *ST3GAL5* variants within highly conserved sialyltransferase motifs in affected individuals with GM3SD.

**Synopsis**

We descibe an affected female with typical features of GM3 synthase deficiency bearing two novel variants in ST3GAL5 that reside in two conserved sialyltransferase motifs and occur in amino acid residues that are strictly invariant across the GT29 sialyltransferase family.

## 1 INTRODUCTION

GM3 synthase deficiency (GM3SD) is caused by biallelic, loss‐of‐function variants in *ST3GAL5*.^1^, ^2^ GM3 synthase catalyzes the formation of the ganglioside GM3 from lactosylceramide (LacCer). GM3 in turn serves as the precursor for the biosynthesis of numerous other glycolipids, including complex gangliosides that are abundantly found in neuronal tissues. GM3 is enriched in lipid rafts at the plasma membrane and been shown to regulate numerous receptor and integrin‐based signaling pathways.^3^, ^4^, ^5^, ^6^, ^7^, ^8^ Loss of GM3 biosynthesis in GM3SD patients is associated with progressive microcephaly due to impaired neurogenesis, hearing loss, dyskinetic movements, seizures, and variable defects in skin pigmentation.^9^, ^10^ The neuronal manifestations of GM3SD reinforce the importance of ganglioside‐mediated processes during neurogenesis, and in the maintenance of proper function of neuronal cell types after development.^6^, ^7^, ^11^, ^12^, ^13^

**FIGURE 1:**
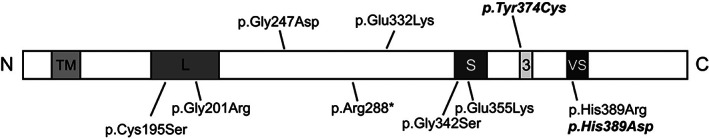
Conserved sialyltransferase motifs and patient variants in the GM3 synthase enzyme. The location of known missense variants and the conserved sialyltransferase motifs in GM3 synthase are depicted. TM, transmembrane region; L, motif L (large); S, motif S (small); 3, motif 3; VS, motif VS (very small). The two variants described in this study are italicized and shown in bold font

GM3SD was originally identified within the Amish population in patients bearing a common homozygous variant (p.Arg288*).^14^ Additional variants in other populations have now been reported, including missense variants within conserved motifs of the GM3 synthase enzyme (Figure 1).^2^, ^12^, ^15^, ^16^, ^17^, ^18^, ^19^, ^20^ These motifs represent conserved functional domains shared across the entire GT29 family of sialyltransferase enzymes.^21^, ^22^ Motif L is thought to contribute to donor substrate binding whereas motif S contains residues that are involved in the binding of both donor and acceptor substrates.^21^ Motif 3 and motif VS contain amino acids that are essential for enzyme activity.^21^ The p.Gly201Arg and p.Cys195Ser variants both reside in motif L, while p.Glu332Lys is found in motif S.^18^ More recently, Heide et al. reported a variant in motif VS (p.His389Arg) in a histidine residue that is strictly invariant across the GT29 family.^16^

In this report, we describe a female with GM3SD with variants in two conserved motifs, including the first reported variant within motif 3 (p.Tyr374Cys). The second allele contains a novel variant (p.His389Asp) within the motif VS of the enzyme. Clinical features in this affected individual are highly consistent with GM3SD. Mass spectrometric analysis of plasma glycolipids demonstrated a striking loss of GM3 in the patient as well as an increase in LacCer and Gb3 that was accompanied by alterations in ceramide chain length. The finding that both alterations occur in highly invariant amino acid residues among GT29 sialyltransferase supports their pathogenicity and highlights the importance of bioinformatic analyses as a component of variant interpretation.

## 2 RESULTS

### 2.1 Clinical summary

The patient, a now 3‐year‐old African‐American and Caucasian female, was first referred to Genetics for evaluation at age 7 months for dysphagia, failure to thrive, electrolyte abnormalities, cortical visual impairment, and developmental delay. She is the first child born to healthy, non‐consanguineous parents. Family history and antenatal history are unremarkable. She was born at full‐term with a birth weight of 3.09 kg (10–25%ile) and length of 45.7 cm (5%ile). The neonatal course was complicated by nuchal cord, feeding difficulties, and temperature instability requiring NICU admission for 6 days. Her clinical picture in relation to known GM3SD findings in summarized in Table 1.

**TABLE 1:** Summary of clinical findings in patient

Previously reported features		Our patient at age 3 years
Growth	Normal growth parameters at birth	X
	Poor growth early in life	X
CNS	Global developmental delays	X
	Microcephaly	X
	Abnormal EEG	X
	Seizures	X
	Dyskinetic movements	X (at 4 months but noted as mild on follow‐up)
	Normal brain MRI in early childhood	X
GI	Feeding difficulties	X
	Reflux	X
	Vomiting	X
	Constipation	X
Musculoskeletal	Scoliosis	
	Contractures	
Audiology	Hearing impairment	
Ophthalmology	Cortical visual impairment	X
	Exotropia	X
	Esotropia	
	Optic nerve atrophy	
Skin	Dyspigmentation	X—per parental report
Behavior	Irritability	X
Sleep	Poor sleep	X

At age 2 months, she was evaluated by Gastroenterology for reflux, gassiness, and constipation at which time she was noted to have moderate malnutrition and reflux. Given limited intake and malnutrition, the patient had a nasogastric tube placed at age 4 months and ultimately required gastrostomy tube placement at age 8 months for persistent dysphagia and poor growth. During inpatient admission at age 4 months for malnutrition and feeding difficulty, the patient had hyponatremia and hyperkalemia and elevated 17‐hydroxyprogesterone concerning for non‐classical congenital adrenal hyperplasia or carrier status for congenital adrenal hyperplasia.

The patient was evaluated by Neurology at age 5 months for developmental delays and lack of visual tracking. MRI of head and orbits at age 6 months was normal. EEG at age 9 months demonstrated multifocal epileptogenicity in the setting of mild, non‐specific generalized cerebral dysfunction. She was diagnosed with cortical visual impairment. At 25 months, family reported concerns with extreme irritability, crying spells, and poor sleep.

Evaluation at age 5 months showed global developmental delay; gross motor skills were commensurate with those of a 2‐month‐old, language skills with those of a 4‐month‐old, adaptive skills with those of a 3‐month‐old, and social skills with those of a 2‐month‐old. At age 14 months, she was beginning to hold her head up while in prone, and beginning to vocalize. At 20 months, she was able to roll both directions, was beginning to bear weight on upper extremities while in prone, was reaching for objects, transferring objects between her hands, and cooing. By 31 months, she was not yet sitting independently but was able to navigate to objects of interest by rolling and stand with assistance of a walker.

On her initial genetics evaluation at age 7 months, her height was 64 cm (*Z* score −1.52), weight was 5.9 kg (*Z* score −2.11), and OFC 40 cm (*Z* score −2.22). Her physical exam was concerning for no observed visual tracking or fixing, for scissoring of the legs when held in suspension and some hypertonicity and straightening of the arms. At her most recent Genetics evaluation at age 25 months, height was 84 cm (*Z* score −0.71), weight 7.83 kg (*Z* score −5.03), and OFC 43.9 cm (*Z* score −2.52). Physical exam was notable for disconjugate gaze, truncal hypotonia, and extremity hypertonia. No pigmentary changes or rashes were identified. She had one parentally reported hyperpigmented macule at birth, a second noted at 6 months of age, and a third hyperpigmented cutaneous change began to develop around age 33 months (File S1).

Brain MRI at 6 months was normal; a rapid MRI at 3 years was nondiagnostic due to motion artifact. EEG at 9 months captured multifocal epileptogenicity in the setting of mild, nonspecific cerebral dysfunction but movement events captured were non‐epileptic.

### 2.2 Diagnostic testing

Metabolic evaluation at age 7 months including plasma amino acids, urine organic acids, acylcarnitine profile, free and total carnitine, lactic acid, and ammonia were normal. Chromosomal microarray returned with negative results. Concerning for non‐classical congenital adrenal hyperplasia (CAH) or carrier status for CAH, CYP11B1, and CYP21A2 sequencing was obtained. Sequencing of *CYP11B1* and *CYP21A2* and PCR RLFP analysis of *CYP21A2* with follow‐up parental testing identified a heterozygous pathogenic variant in *CYP21A2* c.844G>T(p.Val282Leu) and heterozygous variant of uncertain significance in *CYP21A2* c.*13G>A in trans. The involvement of these gene variants was not thought to be relevant to the clinical features in the affected female and clinically interpreted to represent carrier status for CAH. Trio exome sequencing with mitochondrial DNA analysis identified a heterozygous maternally inherited variant of uncertain significance in *ST3GAL5* c.1121 A>G (p.Tyr374Cys) and a heterozygous paternally inherited variant of uncertain significance in *ST3GAL5* c.1165 C>G (p.His389Asp)(NM_003896.3). The p.Tyr374Cys variant is not present in large population databases and p.His389Asp is not observed at a significant frequency in large population cohorts. The analysis of the mitochondrial DNA was non‐diagnostic; a maternally inherited MT‐ND4 m.11420G>A (p.Val221Ile) variant of uncertain significance was identified at ~2% heteroplasmy in blood in the patient.

### 2.3 Variant interpretation

**FIGURE 2:**
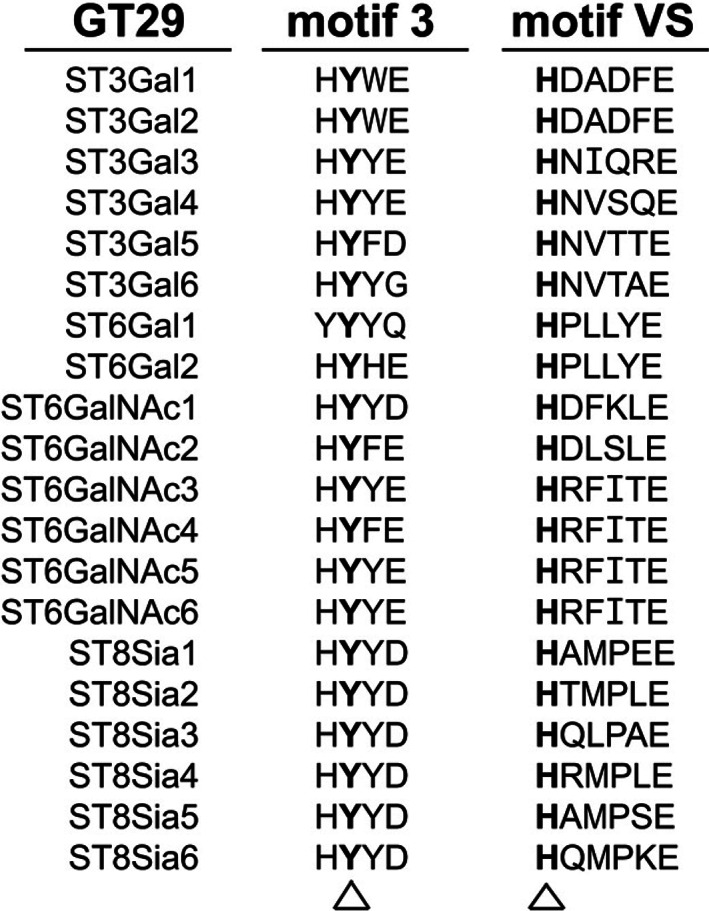
Alignment of motif 3 and motif VS sequences across the GT29 sialyltransferase family. Amino acid sequences for motifs 3 and VS are shown in the different human sialyltransferases within the GT29 family of enzymes. The invariant amino acids in each motif are shown in boldface and denoted by the arrowheads. Figure adapted from Audry et al.^21^

Sequence alignment analysis of the two *ST3GAL5* variants identified in the patient uncovered that both reside in conserved motifs of the GM3 synthase enzyme (motif 3 and motif VS) (Figure 2). These motifs are present in all members of the GT29 family of sialyltransferase enzymes and play a key role in enzymatic activity. Furthermore, the two amino acids altered (Tyr374 and His389) are strictly invariant across the GT29 family, highlighting their importance to enzyme function.

### 2.4 Functional studies

**FIGURE 3:**
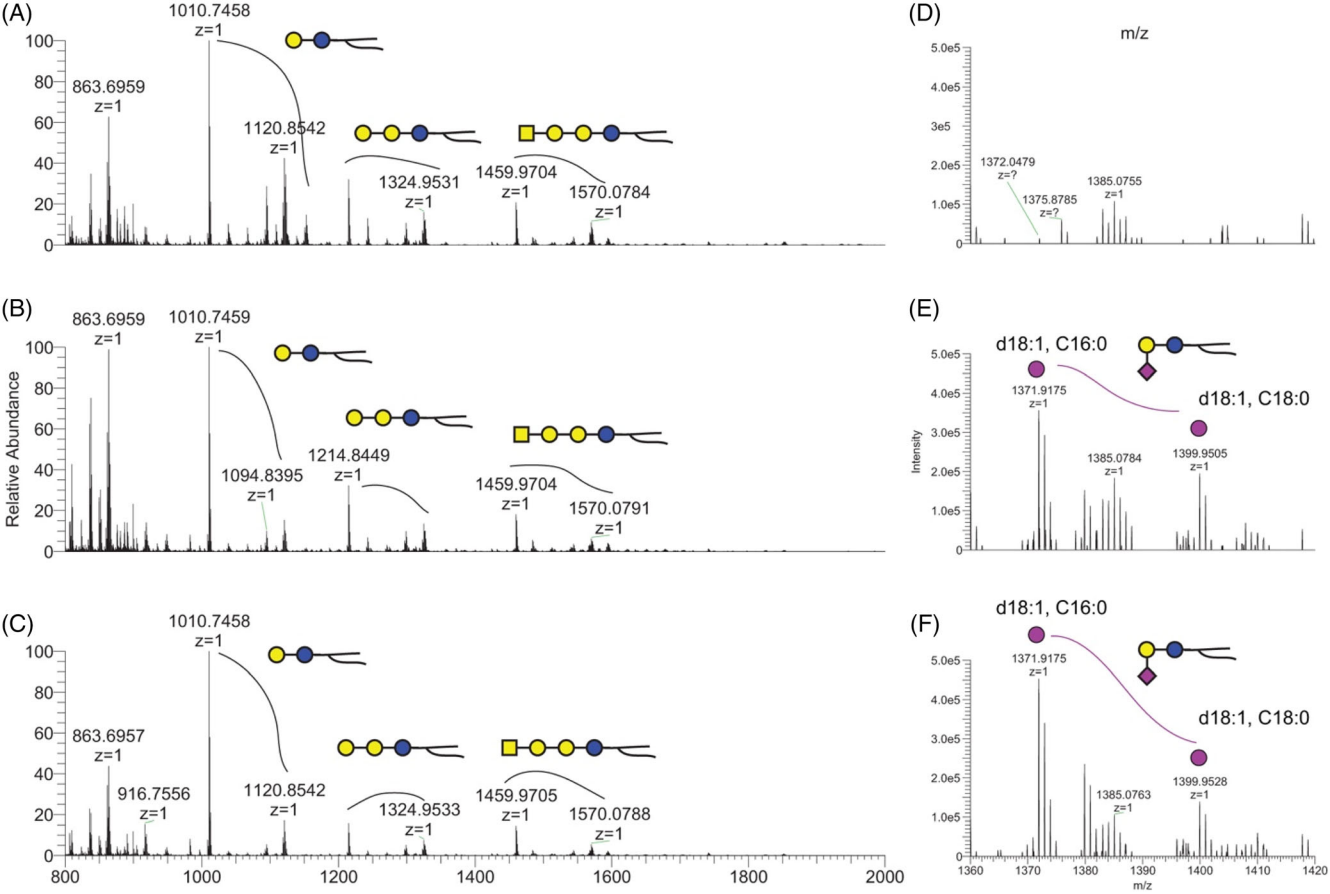
NSI‐MS profiles of plasma glycosphingolipids (GSLs). Full mass spectrometry (MS) spectra are shown for analysis of GSLs prepared from plasma obtained from (A) affected female, (B) father, and (C) mother. Each GSL is associated with multiple peaks that reflect the heterogeneity of the ceramide composition. Magnified MS profiles for the region of GM3 are shown in (D) affected female, (E) father, and (F) mother. GM3 was not detected from the patient's plasma on the full MS profile

**FIGURE 4:**
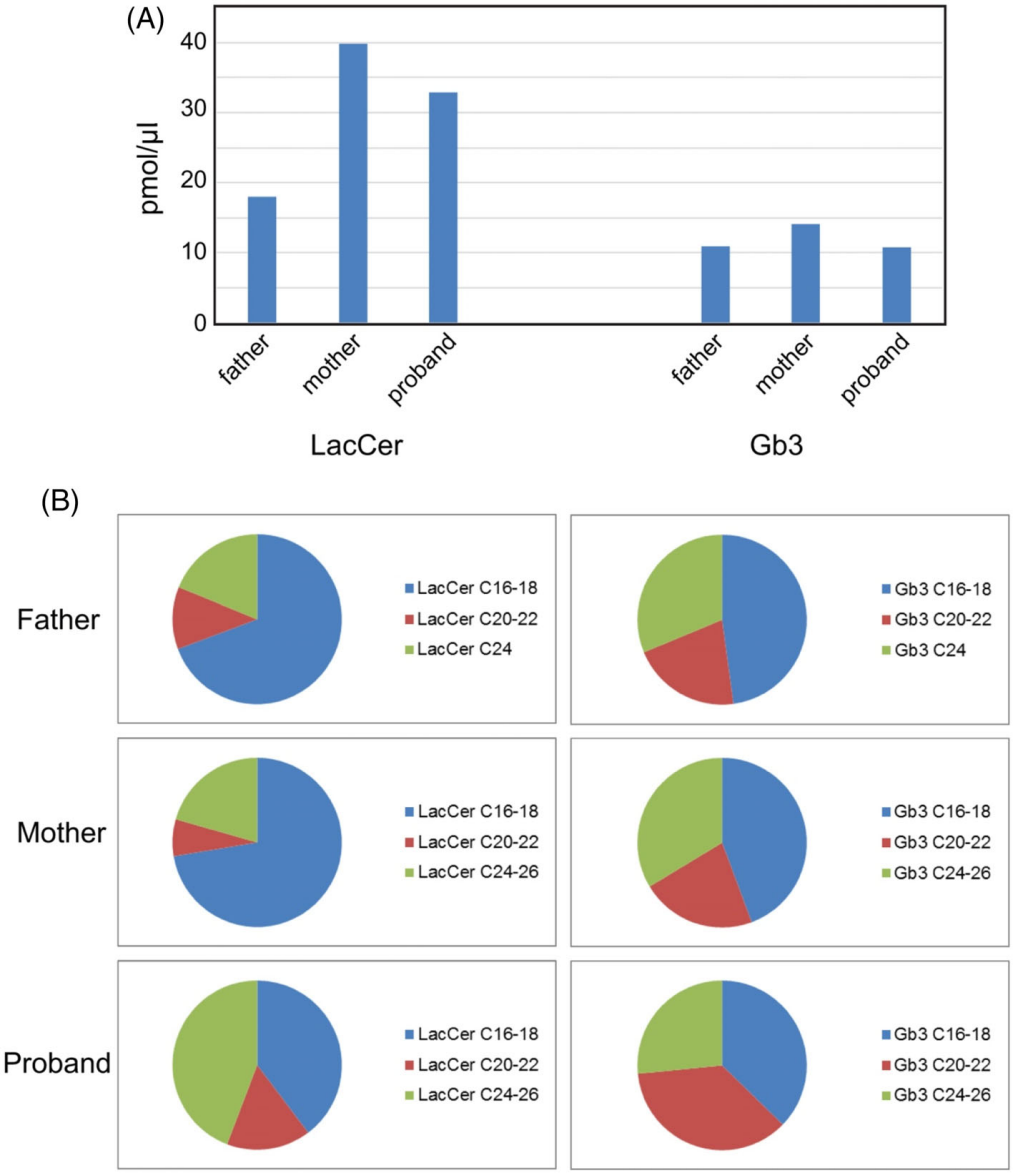
Quantification of LacCer and Gb3 ceramide chain length in the proband and parents. (A) Quantification of LacCer and Gb3 glycolipids in the plasma from parents and proband. (B) Pie charts showing the relative abundance of LacCer and Gb3 molecules with different ceramide chain lengths

Analysis of plasma glycolipids was performed on the proband and both parents using mass spectrometry to confirm that the biallelic variants result in loss of function of the GM3 synthase enzyme. Prior studies have shown that the ratio of GM3 to LacCer is diagnostic for GM3SD.^23^ The results of this analysis are summarized in Figure 3 and clearly show a profound loss of GM3 in the patient (0.5% of either parental control) (see Files S2 and S3). The ganglioside GM1b was detected in the affected female and both parents. LacCer levels were increased in the affected female compared to the parents, although the abundance of this neutral glycolipid was elevated in the mother compared to the father (Figure 4A). The increased LacCer abundance noted in the patient was accompanied by increased ceramide chain length on LacCer (Figure 4B).

**FIGURE 5:**
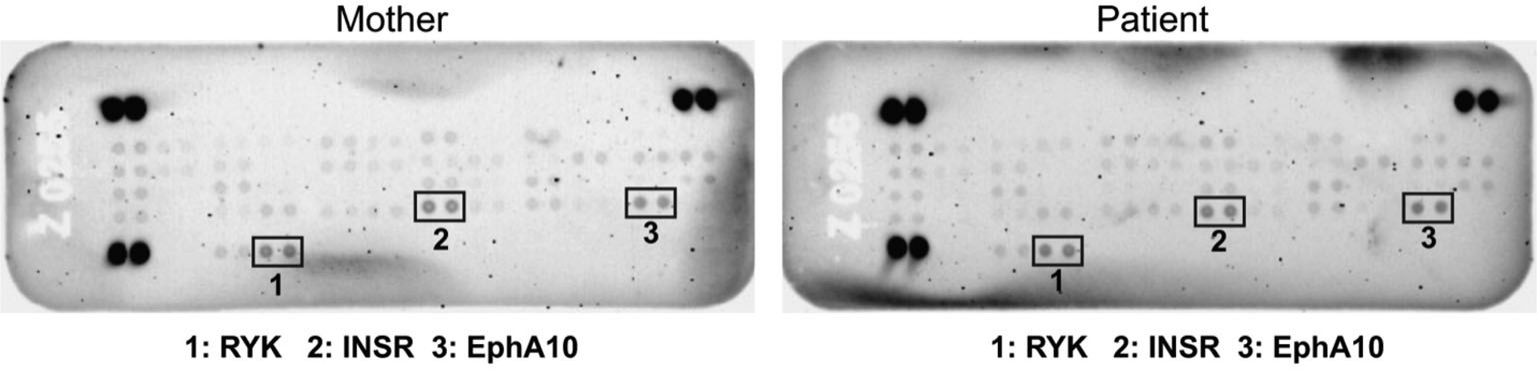
Receptor tyrosine kinase activity is not altered in patient‐derived lymphoblasts. Array‐based analysis of receptor tyrosine kinase activity was performed using EBV‐transformed lymphoblasts from the mother and the affected female. The receptors with the highest activity in these cells—RYK (receptor like tyrosine kinase), INSR (insulin receptor) and EphA10 (ephrin receptor A10)—are boxed. No obvious differences between the mother and patient were detected

As GM3 and other gangliosides are components of lipid rafts and membrane microdomains enriched in signaling receptors, we asked whether any effects on receptor tyrosine kinase (RTK) phosphorylation could be detected in patient cells, supporting the pathogenicity of the variants. We were unable to obtain fibroblasts from the affected female but did generate an Epstein‐Barr virus (EBV)‐immortalized lymphoblast line. Using this cell line, we performed an array‐based analysis of RTK phosphorylation to determine whether altered glycolipid biosynthesis resulted in any abnormality in plasma‐membrane localized RTK activity. As shown in Figure 5, no appreciable differences were observed in the phosphorylation of the receptors with the highest activity on these arrays (RYK, insulin receptor and EphA10), despite the observed alterations in glycolipid profile that were detected in the plasma.

## 3 DISCUSSION

In this paper, we describe a non‐Amish patient with GM3SD bearing two previously unreported variants in *ST3GAL5*. Remarkably both variants occur in amino acid residues within conserved sialyltransferase motifs (motif 3 and motif VS) that are strictly invariant across the GT29 family of sialyltransferases. This observation reinforces the value of bioinformatic analysis of highly conserved domains/motifs for the initial interpretation of variant pathogenicity. Based on the essential role of the conserved tyrosine (Tyr374) and histidine (His389) in the activity of GM3 synthase, we would predict that this affected individual has minimal residual enzyme function, consistent with her age on onset and classic GM3SD phenotype. Although hyperpigmented macules were documented by the parents, there was no evidence of the broader pigmentation defects noted in other GM3SD patients. This further highlights the phenotypic variability that exists across GM3SD patients.

It is unclear whether the ST3Gal5 enzyme made in this affected individual maintains any residual activity but the preponderance of variants within the highly conserved motifs of this sialyltransferase raise the question of whether other more attenuated *ST3GAL5* variants will be identified within human populations bearing less severe clinical manifestations. From a treatment perspective, the pronounced loss‐of‐function effects of these variants indicate that strategies designed to augment residual activity of existing enzyme would have limited benefit, and instead, approaches such as gene replacement or GM3 supplementation are more viable.^9^

A clear increase in the abundance of LacCer with C24‐26 ceramide chains and Gb3 with C20‐22 ceramides was noted in the plasma of the affected female. The basis for increased ceramide chain length in the patient's glycolipids is not known but indicates that alterations in glycolipid biosynthesis in GM3SD may extend beyond the carbohydrate modifications. The mother of the affected female had higher levels of LacCer compared to the father but both were substantially lower than the patient. The ability to confirm the GM3SD diagnosis through the analysis of plasma glycolipids was important for this case as skin biopsies were unable to be obtained from the family.

Our attempt to reveal the functional impact of these variants by broadly analyzing RTK phosphorylation did not uncover any obvious differences in RTK activity in the patient cells, reinforcing the need for functional testing on plasma to determine whether the GM3/LacCer ratios are altered. Although ganglioside profiles in the patient‐derived, EBV‐transformed lymphoblast cells were not analyzed, these cells have been shown to synthesize both neutral and acidic glycolipids whose levels may be altered by GM3 synthase deficiency. It may not be possible to see the functional impact of decreased GM3 levels on receptor activity in this cell type, however, due to the lack of receptors whose activity is modulated by GM3 (e.g., EGFR). Alternatively, it is possible that reduced GM3 abundance impacts receptor tyrosine kinases that are enriched in neuronal tissues, or that the resulting reduction of other complex gangliosides in neurons is responsible for the phenotypic consequences seen in patients. Deeper investigation of GM3SD neuronal pathogenesis using patient‐derived cells is likely to uncover new insights into the cell type‐specific defects of GM3SD.

## 4 MATERIALS AND METHODS

### 4.1 Cell culture

EBV‐transformed lymphoblasts were maintained in RPMI media with 15% fetal bovine serum and antibiotics in a humidified incubator with 5% CO_2_. Cells were subcultured every 2–3 days, and viability determined using trypan blue staining prior to experiments.

### 4.2 Mass spectrometry of plasma glycolipids

Analysis of plasma‐derived glycolipids was performed as described earlier.^23^ Briefly, plasma lipids were extracted with organic solvent and GSLs were enriched on Sep‐pak tC18 cartridge column following saponification. Purified plasma GSLs were permethylated for NSI‐MS analysis as described previously.^24^ GSLs components were identified as singly or doubly charged, sodiated species (M + Na) in positive mode. Peaks for all charge states were summed for quantification. For quantification of permethylated GSLs, permethylated maltotetraose was co‐injected with permethylated GSLs as an external standard.^25^ Graphic representations of monosaccharide residues are consistent with the Symbol Nomenclature for Glycans (SNFG) as adopted by the glycomics and glycobiology communities.

### 4.3 Receptor tyrosine kinase array analysis

Total protein was generated from WT and patient lymphoblasts. Lysates were made using kit manufacturer's Lysis buffer 17 according to manufacturer's protocol (R&D Systems Catalog # ARY001B). After blocking the array blots in Array Buffer for 1 h, 300 μg of protein lysate was incubated on the array blots overnight at 4°C to allow receptors to bind to the immobilized receptor antibodies. Blots were washed three times followed by incubation with an HRP‐conjugated pan phosphotyrosine antibody and allowed to incubate for 1 h on a rocking platform. After washing, chemiluminesent reagents were added for 5 min and the relative phosphorylation of each receptor tyrosine kinase was read using a ChemiDoc system.

## AUTHOR CONTRIBUTIONS

Richard Steet, Michael Tiemeyer, Anna Hurst, Kazuhiro Aoki, and Natasha Rudy were involved in the planning of the study, Kazuhiro Aoki, Cindy Skinner, Lynda Holloway, Amitha Ananth, Anna Hurst, and Natasha Rudy conducted experiments and clinical evaluations, and Richard Steet, Michael Tiemeyer, Anna Hurst, Kazuhiro Aoki, Amitha Ananth, and Natasha Rudy were involved with reporting of the work described in the article.

## CONFLICT OF INTEREST

Natasha Rudy, Kazuhiro Aoki, Amitha Ananth, Lynda Holloway, Cindy Skinner, Anna Hurst, Michael Tiemeyer, Richard Steet declare that they have no conflict of interest related to this manuscript.

## ETHICAL STATEMENT

All procedures followed were in accordance with the ethical standards of the responsible committee on human experimentation (institutional and national) and with the Helsinki Declaration of 1975, as revised in 2000.

## INFORMED CONSENT

Informed consent was obtained from all patients for being included in the study.

## Supporting information


**File S1.** Photos of hyperpigmented macules

**File S2.** TLC of plasma glycolipids

**File S3.** TIM profiles filtered with neutral loss of sialic acid

## Data Availability

The data underlying this article cannot be shared publicly in order to protect the privacy of individuals that participated in the study. The data will be shared on reasonable request to the corresponding author.
