# Supplementary figures and images for: Compound heterozygous variants within two conserved sialyltransferase motifs of ST3GAL5 cause GM3 synthase deficiency
Source: JIMD Rep. 2022 Nov 29;64(2):138–45. doi: 10.1002/jmd2.12353 (PMC9981410; doi:10.1002/jmd2.12353)

**Supplemental file 1- Photos of the hyperpigmented macules on the affected female**


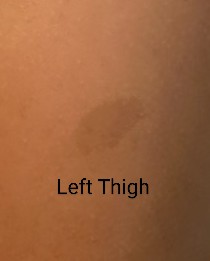

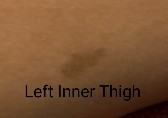


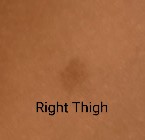

Supplement: Supplementary file 1 — File S1. Photos of hyperpigmented macules [file JMD2-64-138-s001.docx]
