# Supplementary material for: Compound heterozygous variants within two conserved sialyltransferase motifs of ST3GAL5 cause GM3 synthase deficiency
Source: JIMD Rep. 2022 Nov 29;64(2):138–45. doi: 10.1002/jmd2.12353 (PMC9981410; doi:10.1002/jmd2.12353)
Supplement: Supplementary file 2 — File S2. TLC of plasma glycolipids [file JMD2-64-138-s002.docx]

**Supplemental file 2- Thin layer chromatography on plasma glycolipids**

**
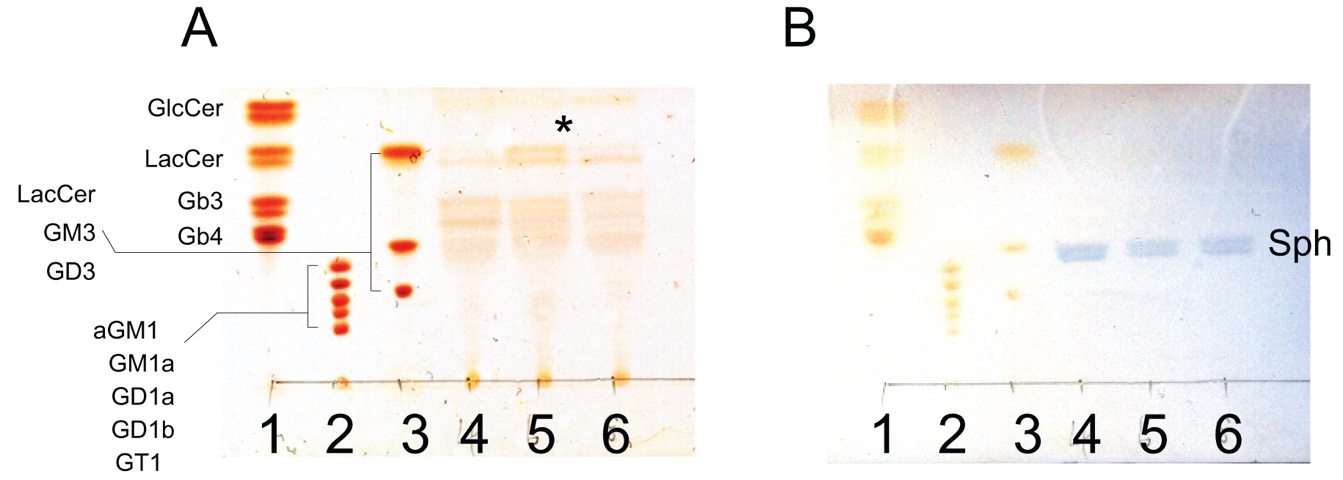
**

Whole lipids were extracted from 300µL of plasma. GSLs were purified on tC18 sep-pak followed by saponification to remove glycerolipids. Purified sphingolipid fraction were reconstituted in 150µL of C:M:W=2:1:0.1, v/v/v, and then 9µL of whole sphingolipid fraction was applied for TLC analysis. A) GSLs stained by orcinol H_2_SO_4_ reagent; B) phosphosphingolipid (PSL) analysis by Dittmer-Lester reagent. The Dittmer-Lester reagent was sprayed on the same TLC plate following detection by orcinol H_2_SO_4_ reagent. Increase of LacCer (*) was observed in the affected female’s GSLs. The major PSL was sphingomyelin (Sph). 1: a mixture of neutral GSLs (Matreya); 2: a mixture of asialogangliosides and monosialogangliosides (Matreya); 3: a mixture of LacCer, GM3 and GD3 (Matreya); 4: GSLs of the patient’s father; 5: GSLs in affected female; 6: GSLs of the patient’s mother.
