# Supplementary material for: Compound heterozygous variants within two conserved sialyltransferase motifs of ST3GAL5 cause GM3 synthase deficiency
Source: JIMD Rep. 2022 Nov 29;64(2):138–45. doi: 10.1002/jmd2.12353 (PMC9981410; doi:10.1002/jmd2.12353)
Supplement: Supplementary file 3 — File S3. TIM profiles filtered with neutral loss of sialic acid [file JMD2-64-138-s003.docx]

**Supplemental file 3- TIM profiles filtered with neutral loss of sialic acid**


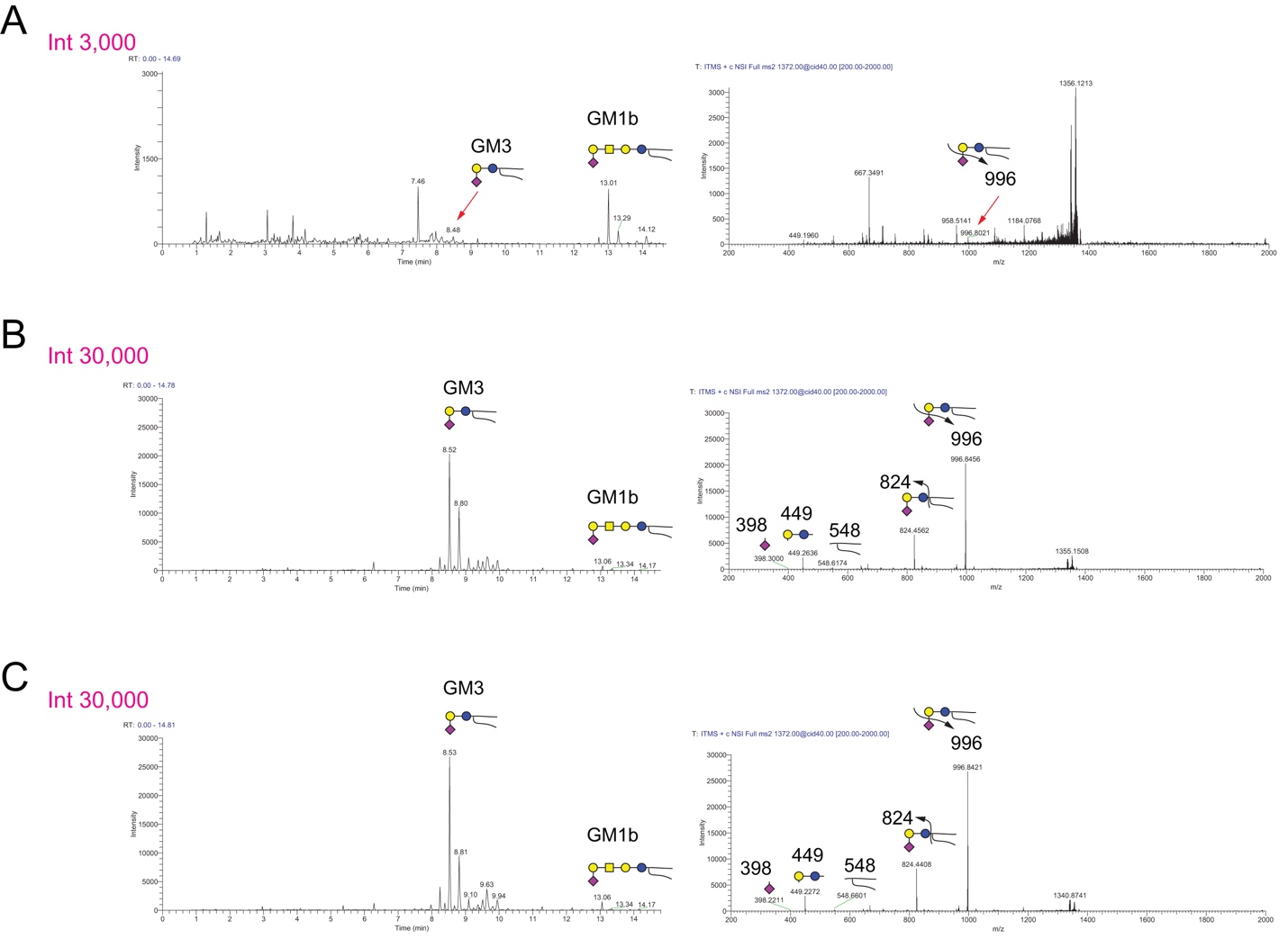


TIM profiles filtered with neutral loss of terminal sialic acid to evaluate the expression of GM3 (left) and MS2 fragmentation pattern of GM3 (right). The permethylated plasma GSL fraction was subjected to total ion mapping (TIM) analysis by nanospray ionization-mass spectrometry (NSI-MS). A) affected female patient, B) father and C) mother. The expression of GM1b are similar in all conditions, however, GM3 was almost absent in the patient's plasma GSLs.
